# Testing and Prediction of the Strength Development of Recycled-Aggregate Concrete with Large Particle Natural Aggregate

**DOI:** 10.3390/ma12121891

**Published:** 2019-06-12

**Authors:** Changyong Li, Fei Wang, Xiangsheng Deng, Yizhuo Li, Shunbo Zhao

**Affiliations:** 1 School of Civil Engineering and Communications, North China University of Water Resources and Electric Power, Zhengzhou 450045, China; fwang@stu.ncwu.edu.cn (F.W.); dxs54371@163.com (X.D.); blackwayne@163.com (Y.L.); 2 International Joint Research Lab for Eco-building Materials and Engineering of Henan, North China University of Water Resources and Electric Power, Zhengzhou 450045, China; 3 Henan Provincial Collaborative Innovation Center for Water Resources High-efficient Utilization and Support Engineering, Zhengzhou 450046, China

**Keywords:** recycled aggregate concrete (RAC), large particle natural aggregate, small particle recycled aggregate, fine recycled aggregate, compressive strength, splitting tensile strength, development prediction

## Abstract

In this paper, a new recycled aggregate concrete (RAC) was produced with composite coarse aggregate and fine recycled aggregate. The composite coarse aggregate was mixed into continuous gradation by large particle natural aggregate with small particle recycled aggregate. To explore the time-dependent developments of the compressive strength and splitting tensile strength of this new RAC, 320 groups of cubic specimens were tested at different curing ages from 3 days to 360 days to measure the compressive and splitting tensile strengths. The amount of large particle natural aggregate varied from zero to 70% in mass of the total coarse aggregate. The water/cement ratio was taken as 0.60, 0.49, 0.41 and 0.36 to represent four strength grades of the RAC at about C20, C30, C40 and C50. Based on the tested results, the curves of the compressive and tensile strengths of the RAC that changed with curing age are plotted, which clearly exhibit that the amount of large particle natural aggregate had a rational range in different strength grades of the RAC which had better aging strength. When the RAC was no larger than C30 with a water/cement ratio of 0.60 and 0.49, the amount of large particle natural aggregate should be no more than 30%; when the RAC was no less than C40 with a water/cement ratio of 0.41 and 0.36, the amount of large particle natural aggregate should be no less than 50%. Along with the general prediction of the strength development of all the tested RAC, the optimal predictive formulas are proposed for the strength development of RAC with a rational amount of natural aggregate. Meanwhile, the strength developments of RAC with a rational amount of natural aggregate are assessed by the time-dependent models proposed by the ACI Committee 209 and CEB-FIP MC 2010.

## 1. Introduction

With the awareness of sustainable social development and strengthened measures to protect natural resources and the environment, the usage of recycled aggregate derived from construction and demolition waste and dismantled concrete structures becomes increasingly important. This leads to a new research area to reuse recycled aggregate as a substitute for natural aggregate to produce a new concrete, commonly called recycled aggregate concrete (RAC) [1,2,3]. In the abundant literature about RAC, many researches took conventional concrete as the reference to mainly concern the effect of the replacement of coarse natural aggregate with coarse recycled aggregate in the same grading, where the replacement rate was usually taken as an important parameter to reflect the influence of recycled aggregate on the performance of RAC [4,5,6,7,8,9,10,11]. Due to the difference of the mix proportion design of RAC, different research results were concluded. For example, Lotfi et al. [4] discovered that the properties of RAC were less influenced by the replacement rate of recycled aggregate, while they were directly affected by the grade of cement and the water/cement ratio; Yao et al. [10] revealed that when the replacement rate of coarse recycled aggregate with a particle size of 19–26.5 mm varied from 0% to 100%, only the cases of 0% and 60% led to the maximized compressive and splitting tensile strengths of RAC; Liu et al. [11] explained by numerical analyses that the compressive and flexural strengths of RAC decreased gradually with the increase of the amount of coarse recycled aggregate, and the flexural strength decreased with the increasing maximum particle size of coarse recycled aggregate. Therefore, the structural behaviors of RAC members are generally weaker in comparison to those of structures made of natural aggregate concrete, although RAC can be used through proper design from the view point of loading capacity behavior. This can be attributed to ignoring the special morphology of recycled aggregate with a different particle size.

Different from natural aggregate, recycled aggregate has peculiarities characterized by its rough surface with a certain amount of attached old cement mortar, low density, and a high and quick rate of water absorption. This happens to be related to the particle size, as large particle recycled aggregate had a more adverse effect on the performance of RAC compared with small particle recycled aggregate [2,3,4,12,13,14,15]. That is, with the increase of particle size, the probability of defects increased gradually in aggregate and the bond properties of the interface between the new cement mortar and the recycled aggregate were weakened. For example, Seo and Choi [12] revealed that when the size of the recycled aggregate was around 10 mm, certain angularity and adequate old cement mortar that attached to the recycled aggregate surface contributed to the bond quality between the aggregate and cement mortar. Qiao et al. [16] demonstrated that the compressive strength of RAC firstly increased and then decreased with the increasing particle size of the coarse recycled aggregate, and reached the highest when the particle size grading was 5~20 mm. Li and Gao [17] discovered that with the increasing maximum particle size of coarse recycled aggregate, the compressive strength of RAC decreased; especially in the case of a lower water/cement ratio, the adverse effect of particle size on the compressive strength became more obvious. Therefore, the recycled aggregate should be reused in small particle size [18,19]. However, from the perspective of concrete technology, in terms of achieving the same target compressive strength and workability for structural concrete, adjustments to the mix proportion should be made within the limit of maximum water/binder ratio considering the concrete durability [20,21]. With the appropriate mix design [22,23], reasonable mineral admixtures [22,24,25,26], pre-soaked recycled aggregate and a proper mixing process [24,25,27,28,29], or the modification of the coarse recycled aggregate surface [30,31], the performance of RAC can be improved to equal or greater than that of the reference concrete with natural aggregates. 

Meanwhile, compared with natural sand, fine recycled aggregate had very different characteristics with angular and irregular particles, high mortar content, non-uniform morphology containing a mixture of C-S-H and CH formations, low density and high water absorption [2,3,32,33]. In some instances, RAC with fine recycled aggregate presented worse workability, larger shrinkage and lower mechanical performance than the reference concrete with natural sand [2,3,34,35,36]. However, by using partial fine recycled aggregate replacing natural sand, adding the super-plasticisers, the same or exceeding performances of RAC with fine recycled aggregate was reached [14,19,35,36,37,38]. Even though in the case of RAC only having recycled fine and coarse aggregate, the required basic performances of RAC can be obtained by comprehensively using the methods of pre-soaking recycled aggregate, adjusting the mix proportion and admixing super-plasticisers [19,34,38,39].

Based on the review of current studies and the engineering experience of manufacturing recycled aggregate, an innovative concept was gradually formed in our research procedure. If the particle size of recycled aggregate was made less than that of the mother aggregate in the waste concrete, the defects of the coarse recycled aggregate could be eliminated as much as possible, especially in the removal of the attached old cement mortar [13,14,18,19]. The grading of the coarse aggregate for RAC can be perfectly composited by using large particle natural aggregate and small particle recycled aggregate [38,40]. At the same time, the fine recycled aggregate is still used as the only fine aggregate to get the best benefits of the engineering application of RAC [19,38,39,41]. Comprehensively, a new RAC was innovated, in which the continuous grading coarse aggregate was composited by large particle natural aggregate with small particle recycled aggregate, and only fine recycled aggregate was used as the fine aggregate [40,42,43]. 

Due to the fact that the long-term strength of concrete is essential to the reliability of concrete structures, it is necessary to investigate the strength development of the RAC to be used as a structural material. In this paper, the experimental study was carried out based on the experience of previous researches [39,44,45]. Four strength grades of the RAC represented by different water/cement ratios were prepared, and each of them were made of four groups with different amounts of natural aggregate. The cubic specimens of RAC were fabricated as 320 groups and tested 10 times at different curing ages up to 360 days to get the compressive and splitting tensile strengths. Based on the experimental results, the rational amount of large particle natural aggregate in RAC with different strength grade was determined and the predictive equations of the strength development can be proposed. Finally, the strength developments of RAC with a rational amount of natural aggregate will be assessed by the time-dependent models specified in the codes of ACI and CEB-FIP.

## 2. Materials and Methods

### 2.1. Raw Materials

**Figure 1:**
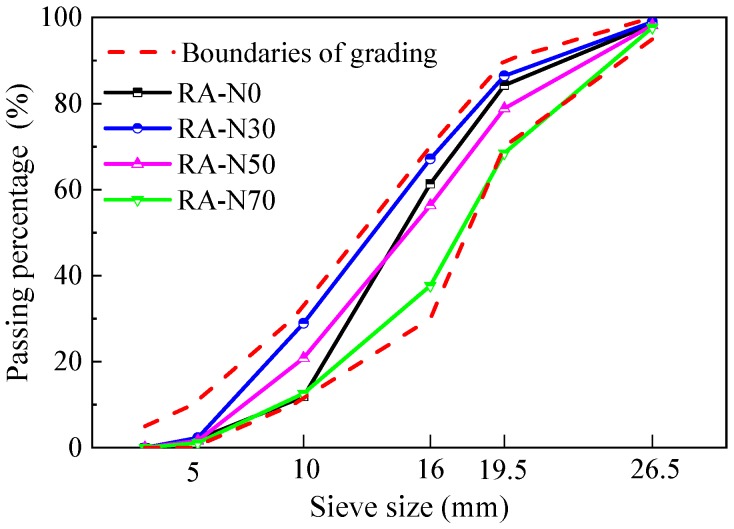
Grading curves of the aggregates used.

**Figure 2:**
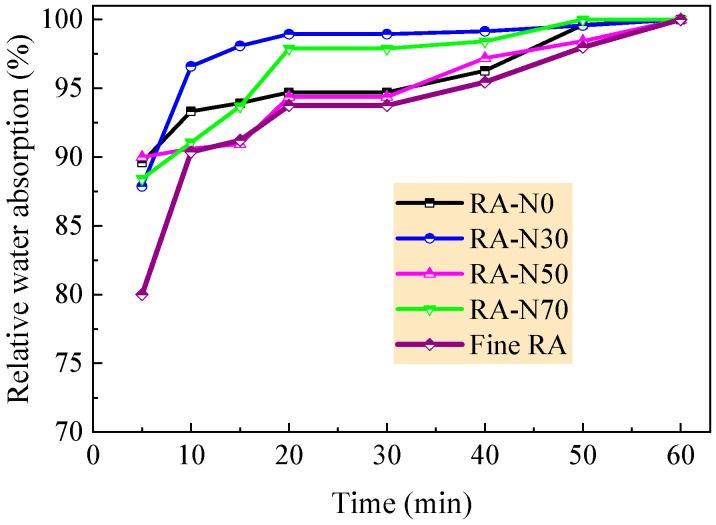
Relative water absorption of aggregates within 1 h.

The coarse recycled aggregate was produced from the waste concrete beams tested previously in the lab and was screened into the series of 5–10 mm, 10–16 mm, 16–20 mm and 20–25 mm according to the requirements of China code GB/T25177 [46]. For the RAC with large particle natural aggregate, the coarse aggregate was composited with the small particle recycled aggregate in sizes of 5~10 mm and 10~16 mm and the large particle natural aggregate in sizes of 16–20 mm and 20–25 mm. The amount in mass of natural aggregate to total aggregate was defined as 30%, 50% and 70%, respectively. The corresponding RACs were marked as RAC-N30, RAC-N50 and RAC-N70. The amounts were determined in accordance with the requirement that in the 5–25 mm continuous particle gradation of coarse aggregate for RAC and conventional concrete [46,47], 30–70% of aggregate should be used with a particle size no less than 16 mm. Meanwhile, the RAC without natural aggregate was marked as RAC-N0, which was made of coarse recycled aggregate with 5–25 mm particles. Table 1 presents the physical and mechanical properties of coarse aggregates. The grading curves of aggregates are exhibited in Figure 1, which are in the boundaries of grading specified in China standards GB/T 25177 and JGJ52 [46,47]. The water absorption curves of aggregates within 1 h are represented in Figure 2. With the increasing amount of natural aggregate, the coarse aggregates had an increased density with lower moisture content, water absorption and crushed index. This is reasonable due to the lower water absorption of natural aggregate.

The recycled aggregate was produced from the waste concrete specimens tested in the lab. The particle size of the coarse recycled aggregate was less than the mother aggregate in the waste concrete. The fine aggregate was the byproducts accompanied with the production of the coarse recycled aggregate with a particle size less than 5 mm after being severed in grading as the requirements of GB/T25176 [48]; the properties that were measured are listed in Table 2. 

The ordinary silicate cement in strength grades of 42.5 and 52.5 was used as the binder; the physical and mechanical properties are listed in Table 3. Other raw materials included tap water and the commercially available high-performance polycarboxylic acid water-reducer with a water reducing rate of 15%.

### 2.2. Mix Proportions of RAC

Due to the difference of properties between recycled aggregate and natural aggregate, the mix proportion of the RAC was designed based on the absolute volume method [19,49]. The water/cement ratio and the amount in mass of natural aggregate were considered as the main factors. To obtain good workability and expected strength of the RAC, the sand ratio of fine recycled aggregate to total amount of fine and coarse aggregate used in this experiment was determined based on previous studies [13,19,42] and was adjusted after trial mixing. Considering the higher water absorption of recycled aggregate, the additional water was added appropriately based on the water absorption test results, as presented in Figure 2, to get the same condition of surface-drying saturated aggregates as per the specification of China standard JGJ55 [49]. After adjustment, the mix proportions of the RAC were determined, as presented in Table 4.

### 2.3. Specimen Preparation and Testing

The single-horizontal-shaft forced mixer was used to mix the fresh mixture of the RAC. The aggregates were firstly pre-soaked by half dosage of water in the mixer for 10 min to basically meet the requirement of the surface-drying saturated condition, then the cement and the residual water as well as the super-plasticizer were added and mixed for 3 min.

Cubic specimens with dimension of 100 mm were cast in moulds, compacted on the vibration table and covered with polyethylene sheet on the casting surface for 24 h. After that, they were demoulded and cured at the standard curing room temperature of 20 ± 2 °C and *RH* over 95% [50]. Details of curing age for tests are presented in Table 5.

The tests were carried out on the 1000 kN electric-hydraulic servo-testing machine (Jinan Testing Machine Co. Ltd., Jinan, China). The loading process was in accordance with the specification of China code GB/T 50081 [50] and the tested compressive strength and splitting tensile strength were corrected by multiplying the reduction coefficients 0.95 and 0.85, respectively, as the values of standard cubic specimen in dimension of 150 mm.

## 3. Results and Discussion

### 3.1. Properties of Fresh RAC

**Figure 3:**
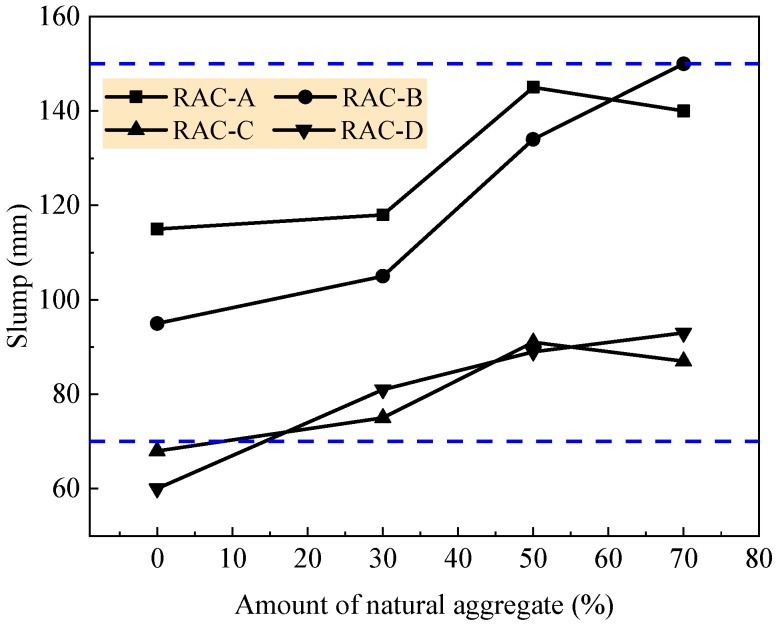
Slump of fresh RAC.

The workability of fresh RAC was measured by using the slump cone test [51]. In this test, the maximum particle size of the coarse aggregate was 25 mm. In order to meet the flowability of fresh concrete from plastic to flowing, the slump was designed in a range of 70–150 mm. Each slump value reported in this paper was the average of three readings obtained from each trial in the same conditions. The slump of fresh RAC is presented in Figure 3. Generally, the fresh RAC had good cohesiveness and water retention within the slump measured from 60 mm to 150 mm. The slump of fresh RAC increased with the increasing amount of natural aggregate. This is due to plenty of cement paste among the particles of aggregates, as less amount of cement paste was needed to be wrapped on the natural aggregate with good particle morphology [13,19,42]. Comparatively, fresh RACs with a large water/cement ratio of 0.6 and 0.49 had higher flowability than those with small water/cement ratios of 0.41 and 0.36. This is mainly due to the higher dosage of water, as presented in Table 4, which was used as per the water dosage for conventional concrete having only natural aggregate specified in China standard JGJ55 [49], which related to the large particle size of natural aggregate and the dosage of water-reducer.

### 3.2. Development of Compressive Strength

**Figure 4:**
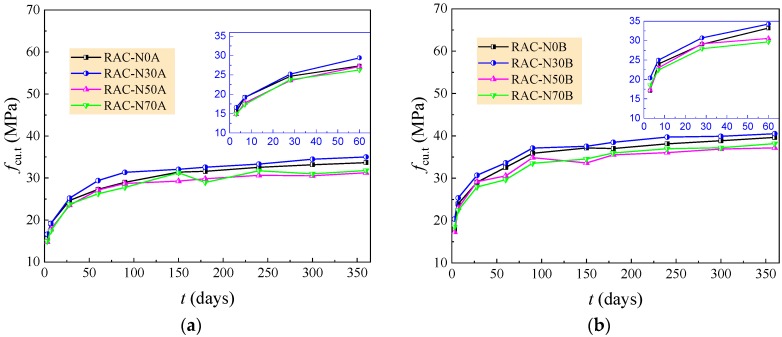

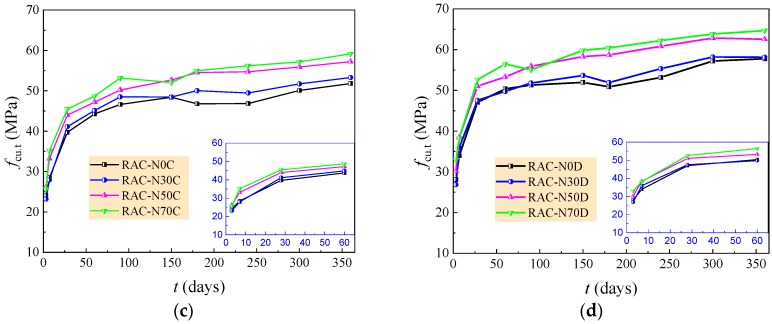
Changes of compressive strength of the RAC with curing age. (**a**) *w/c* = 0.60; (**b**) *w/c* = 0.49; (**c**) *w/c* = 0.41; (**d**) *w/c* = 0.36.

Figure 4 presents the changes of compressive strength *f*_cu,t_ of the RAC with curing age *t*. The development of compressive strength went through three periods of rapid growth before *t* = 28 days, fast increase during *t* = 28–90 days and steady increase after *t* = 90 days. This corresponds to the hydration process of cement in the mixture [44,52,53,54]. With the reduction of the water/cement ratio, the first period of rapid growth of compressive strength became obvious. This is due to the influence of the water/cement ratio, as the cement in a different strength grade had a certain impact on the hardening procedure. The higher fineness of 52.5 grade cement provides a larger interface area of the cement particles for rapid hydration, and the higher early strength of 52.5 grade cement benefits the early strength of the concrete [39,44,45].

In Figure 4, the amount of natural aggregate had a certain effect on the compressive strength of the RAC, which was mainly related to the *w*/*c* [19,38,39,40]. When *w/c* = 0.6 and 0.49, the RAC with 0% and 30% natural aggregate had higher compressive strength, while those with 50% and 70% natural aggregate had lower compressive strength. When *w/c* = 0.41 and 0.36, the RAC with 50% and 70% natural aggregate had higher compressive strength, although 30% natural aggregate in the RAC also benefited the compressive strength. This reflects the different functions of hydrated cement, coarse aggregate and their interfaces in the RAC. For the RAC with *w/c* = 0.6 and 0.49, the compressive strength was lower and mainly relied on the strength of the set cement and the bond strength of the interfaces. The rough surface with a large water absorption of recycled aggregate provided a large water storage capacity to cement hydration with the gradually released water [16,39,40]. In this case, the coarse aggregate had less of an effect on the compressive strength of the RAC as the main failure of RAC took place within the hardened set cement and/or along the interfaces with weakened bond performance [12,15]. For the RAC with *w/c* = 0.41 and 0.36, the failure took place due to the peeling off and/or splitting of the coarse recycled aggregates. The skeleton strength of the coarse aggregate became important in the contribution to the compressive strength of the RAC. With the increasing amount of large particle natural aggregate, the crush index of the coarse aggregate decreased from 14.7% to 13.2% (see Table 1). In this case, combined with good bond property of interfaces with a lower water/cement ratio, the benefit of large particle natural aggregate turned up on the compressive strength of the RAC.

Considering the discrete characters of the development of compressive strength, the ratio of compressive strength of the RAC with/without natural aggregate at curing age *t* ≥ 28 days was computed. The average and variation coefficient of the ratios are listed in Table 6. The ratio over 1.0 represents the positive effect of large particle natural aggregate on the compressive strength of the RAC. It can be concluded that the rational amount of large particle natural aggregate is 30% for the RAC with *w/c* = 0.6 and 0.49, and 50–70% for the RAC with *w/c* = 0.41 and 0.36.

### 3.3. Development of the Splitting Tensile Strength

**Figure 5:**
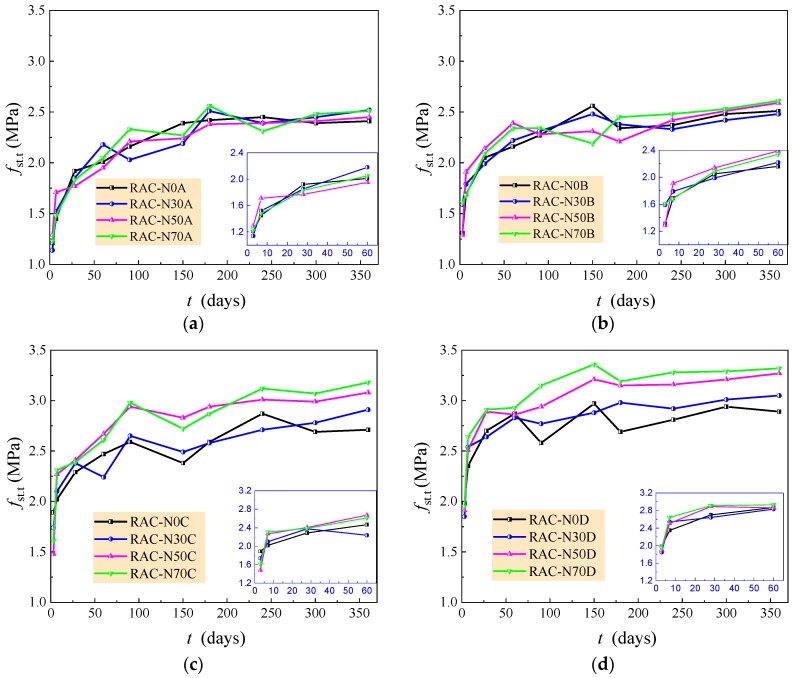
Changes of splitting tensile strength of the RAC with curing time. (**a**) *w/c* = 0.60; (**b**) *w/c* = 0.49; (**c**) *w/c* = 0.41; (**d**) *w/c* = 0.36.

Figure 5 shows the changes of splitting tensile strength *f*_st.t_ of the RAC with curing age *t*. The splitting tensile strength had a similar development to the compressive strength as discussed above, except there was no obvious influence of the amount of natural aggregate when *w/c* = 0.6 and 0.49. The main reason for this difference was the different failure mechanism of the RAC under compression and splitting tension. The failure under splitting tension of the RAC mainly depended on the bond of the interface between the coarse aggregate and set cement. Due to the beneficial effect of recycled aggregate with old cement mortar and a rough surface, the bond performance could be equivalent to that natural aggregate in the condition of the RAC with a larger water/cement ratio [12,13,14,15,39,44]. This results in the independence of tensile strength of the RAC to the composite of coarse recycled and natural aggregates. In the case of the RAC with *w/c* = 0.41 and 0.36, the tensile strength of the RAC tends to increase with the increasing amount of large particle natural aggregate. This results from the relative perfect surface of the natural coarse aggregate, which provides good bond performance between set cement and aggregates [15,16,17].

Considering the discrete characters of the development of splitting tensile strength, the ratio of splitting tensile strength of the RAC with/without natural aggregate at curing age *t* ≥ 28 days was computed. The average and variation coefficient of the ratios are listed in Table 7. The ratio over 1.0 represents the positive effect of large particle natural aggregate on tensile strength of the RAC. The tensile strength of the RAC with *w/c* = 0.6 and 0.49 increases slightly with the increasing amount of large particle natural aggregate, while that of the RAC with *w/c* = 0.41 and 0.36 increases clearly with the increasing amount of large particle natural aggregate.

## 4. Prediction of Strength Development

### 4.1. Compressive Strength

In the meso-level, concrete can be regarded as the composites of inert aggregates with various sized particles scattered in a homogeneous matrix of hardened cement gel. With the optimal design of the types and composite of aggregates, the compressive strength of the concrete is related to the volume fraction of the cement gel and the compressive property of the cement. The compressive property of the set cement can be represented as the cement compressive strength *f*_ce_ at 28 days. The volume fraction of the cement gel is positively related with the cement slurry density in fresh concrete. Referring to the cement compressive strength model [42], the cement slurry density in fresh concrete can be expressed as *V*_c_/(*V*_c_ + *V*_w_ + *V*_a_), where *V*_c_, *V*_w_ and *V*_a_ are the volumes of cement, water and air, respectively. Meanwhile, the kinetics term (*d(t)*) should be inserted in the compressive strength equation to express the effects of the aggregate bond effect and ceiling effect with curing age. Therefore, the concrete compressive strength model at *t* days was built as,

(1) $$f_{\mathrm{cu}.t}=a\;f_{\mathrm{ce}}\left(d(t)+{\left(V_{c}/(V_{c}+V_{w}+V_{a})\right)}^{b}\right)$$

where *a* and *b* are regression coefficients determined by the test’s data.

As mass is commonly used in engineering, taking water density as 1000 kg/m^3^ and cement density as $\rho_{c}$, ignoring the volume of air and translating volume density *V*_c_/ (*V*_c_ + *V*_w_) to mass ratio (i.e., water/cement ratio) *w*/*c* [44]. Due to the logarithmic development of compressive strength, replacing *d(t)* by *k*lg(*t*/28). Equation (2) can be transformed as,

(2) $$f_{\mathrm{cu}.t}=a\;f_{\mathrm{ce}}\left(k\mathrm{lg}(t/28)+{\left(1+0.001\rho_{c}w/c\right)}^{-b}\right)$$

After being fitted with test data of compressive strength at 28 days of this study, *a* = 2.83 and *b* = 1.60.

**Figure 6:**
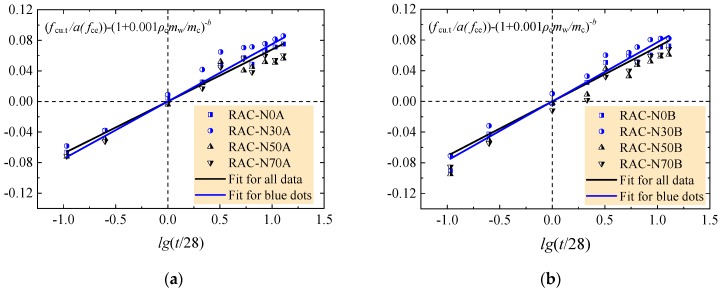

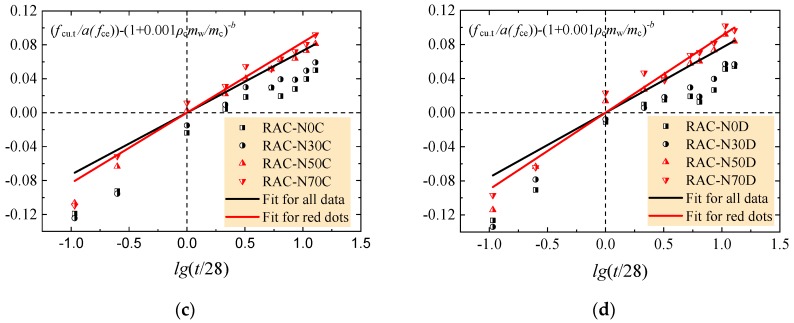
Analysis of the tested data about compressive strength. (**a**) *w/c* = 0.60; (**b**) *w/c* = 0.49; (**c**) *w/c* = 0.41; (**d**) *w/c* = 0.36.

To get the general development regulation of the RAC, taking the tested values of each trials as *f*_cu.t,_ and using lg(*t*/28) as the x-axis and (*f*_cu.t_/(a*f*_ce_))−(1 + 0.001*ρ*_c_*w/c*)^−b^ as the y-axis, the coefficient *k* can be obtained by the fitting analysis exhibited in Figure 6, and the values are presented in Table 8.

**Figure 7:**
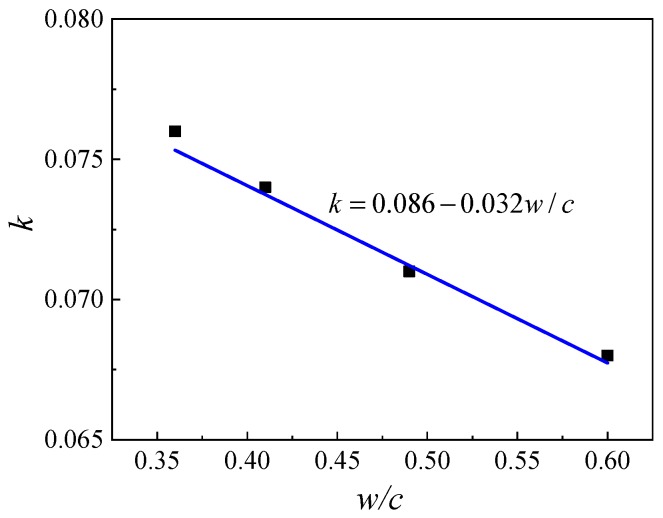
Relation of coefficient *k* with *w/c*.

The relationship between *k* and *w*/*c* can be fitted as linear as presented in Figure 7.

Therefore, Equation (2) can be rewritten as,

(3) $$f_{\mathrm{cu}.t}=2.83\;f_{\mathrm{ce}}\left((0.086-0.032w/c)\mathrm{lg}(t/28)+{\left(1+0.001\rho_{c}w/c\right)}^{-1.60}\right)$$

**Figure 8:**
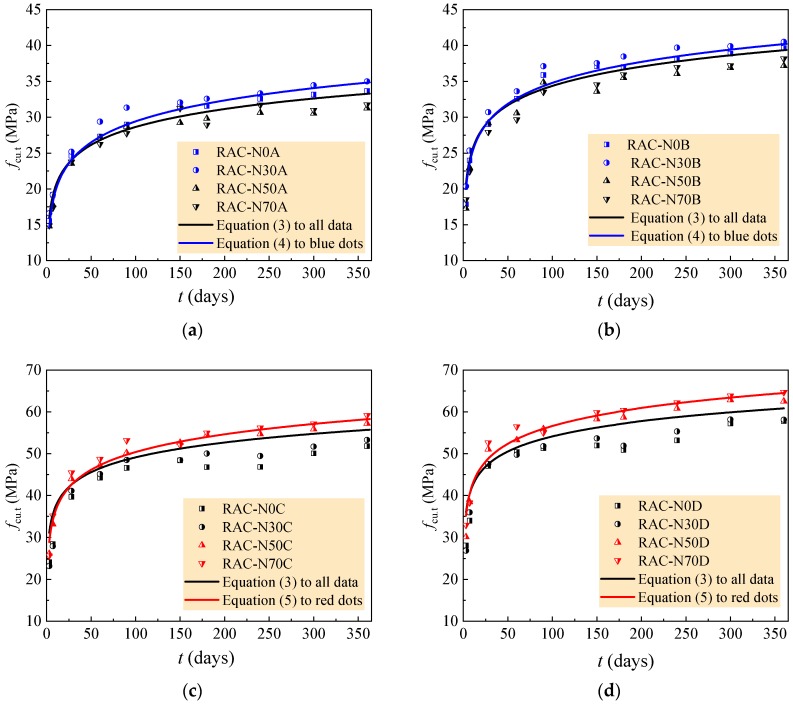
Comparison of calculated values and tested values by Equations (4), (5) and (6). (**a**) *w/c* = 0.60; (**b**) *w/c* = 0.49; (**c**) *w/c* = 0.41; (**d**) *w/c* = 0.36.

The comparison of the experimental data to curves of Equation (3) are exhibited in Figure 8, and the statistical results for each group of RAC are listed in Table 9. Safe prediction of strength development is given out when the ratio of tested to calculated values is larger than 1.0. It can be seen from Table 10 that Equation (3) was safe for the RAC with 0–30% natural aggregate in the case of *w/c* = 0.60 and 0.49, and for RAC with 50–70% natural aggregate in the case of *w/c* = 0.41 and 0.36. In other cases, the calculated values were greater than the tested data from 1.89% to 7.24%.

In order to accurately predict the compressive strength of the RAC with a rational amount of large particle natural aggregate, as exhibited in Figure 6, the coefficient *k* was fitted separately for the RAC of N0A and N30A, N0B and N30B, N50C and N70C, N50D and N70D. The value was 0.075, 0.077, 0.083 and 0.090, successively. Therefore, it takes 0.076 for the RAC with *w/c* = 0.60 and 0.49, and 0.086 for the RAC with *w/c* = 0.41 and 0.36. Equation (2) can be rewritten as Equations (4) and (5), respectively.

(4) $$f_{\mathrm{cu}.t}=2.83\;f_{\mathrm{ce}}\left(0.076\mathrm{lg}(t/28)+{\left(1+0.001\rho_{c}w/c\right)}^{-1.60}\right)$$

(5) $$f_{\mathrm{cu}.t}=2.83\;f_{\mathrm{ce}}\left(0.086\mathrm{lg}(t/28)+{\left(1+0.001\rho_{c}w/c\right)}^{-1.60}\right)$$

The comparison of experimental data to curves of Equations (4) and (5) are also exhibited in Figure 8, and the statistical results for each group of the RAC are listed in Table 10. These represent that more reliable predictions are provided for the compressive strength development of the RAC. 

### 4.2. Splitting Tensile Strength

The splitting tensile strength *f*_st.28_ is usually predicted by cubic compressive strength *f*_cu.28_ with the expression of Equation (6),

(6) $$f_{\mathrm{st}.28}=cf_{\mathrm{cu}.28}{}^{d}$$

where *c* and *d* are coefficients dependent on test data. 

**Figure 9:**
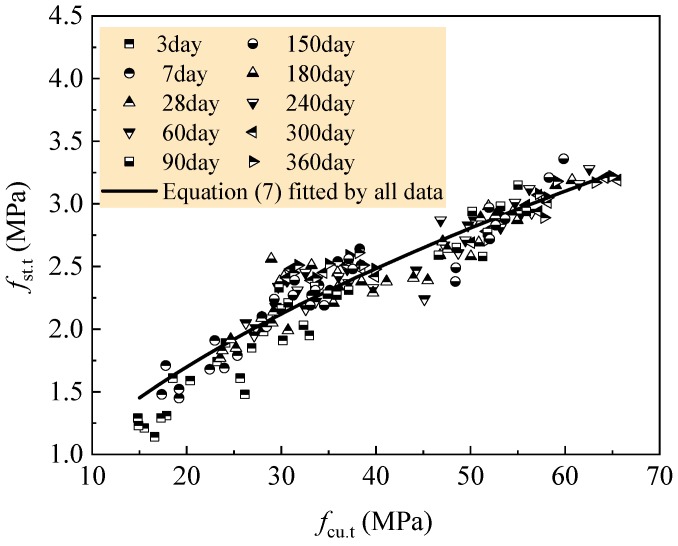
Unified fitness of splitting tensile strength *f*_st.t_ with compressive strength *f*_cu.t_.

Based on previous studies [39,45,52], Equation (6) can also be applied to the prediction of splitting tensile strength of concrete at any curing age of *t* days. Therefore, the general development regulation of tensile strength of the RAC is statistically analyzed by all of the test data. The unified values are *c* = 0.329, *d* = 0.548 and *R* = 0.945. Equation (6) can be rewritten as Equation (7), and good fitness of test data with predicted values can be calculated, as exhibited in Figure 9.

(7) $$f_{\mathrm{st}.t}=0.329f_{\mathrm{cu}.t}{}^{0.548}$$

**Figure 10:**
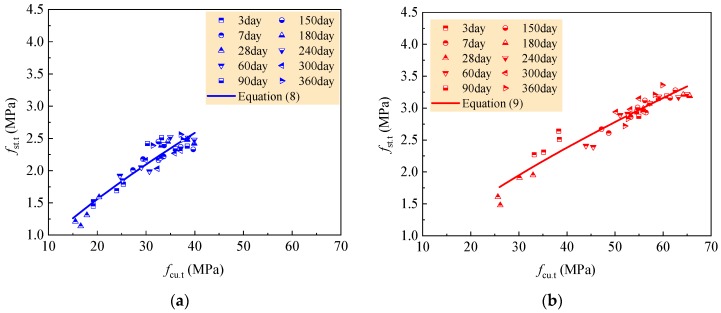
Fitness of splitting tensile strength *f*_st.t_ with compressive strength *f*_cu.t_: (**a**) N0A, N30A, N0B and N30B; (**b**) N50C, N70C, N50D and N70D.

Meanwhile, accurate prediction for the RAC of N0A, N30A, N0B and N30B can be done by taking *c* = 0.173, *d* = 0.733 with *R* = 0.882; the accurate prediction for the RAC of N50C, N70C, N50D and N70D can be done by taking *c* = 0.182, *d* = 0.697 with *R* = 0.920. Equation (6) can be rewritten as Equations (8) and (9), respectively. Figure 10 presents the fitness of test data with the curves of these two equations.

(8) $$f_{\mathrm{st}.t}=0.173f_{\mathrm{cu}.t}{}^{0.733}$$

(9) $$f_{\mathrm{st}.t}=0.182f_{\mathrm{cu}.t}{}^{0.697}$$

**Figure 11:**
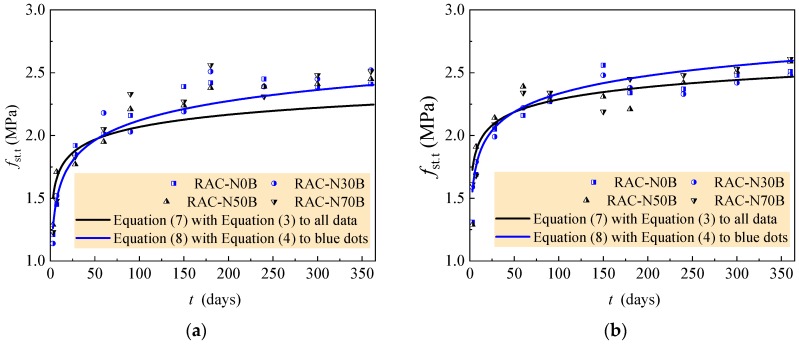

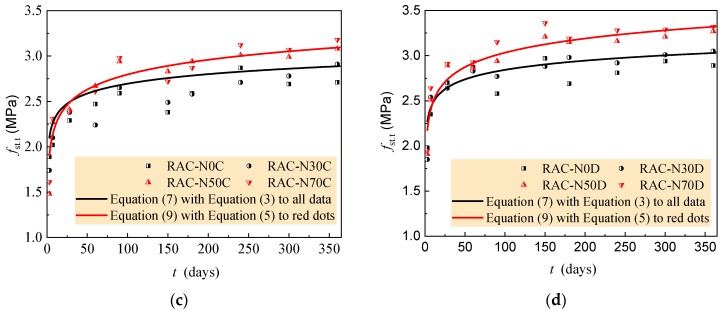
Comparison of test data to curves of equations. (**a**) *w/c* = 0.60; (**b**) *w/c* = 0.49; (**c**) *w/c* = 0.41; (**d**) *w/c* = 0.36.

Therefore, the tensile strength development of all tested RAC can be predicted by Equation (7) combined with Equation (3). Figure 11 exhibits the fitness of test data with predicted curves. Table 11 presents the statistical results of the ratios of tested to computed values for each group of RAC. The ratio, on average, of no less than 1.0 means a safe prediction. Except for the safe prediction, others have the bigger prediction from 3.81% to 5.55%.

The tensile strength development of the RAC with 0–30% natural aggregate in the case of *w/c* = 0.60 and 0.49 can be predicted by Equation (8) combined with Equation (4), and that of the RAC with 50–70% natural aggregate in the case of *w/c* = 0.41 and 0.36 can be predicted by Equation (9) combined with Equation (5). The comparisons of predicted curves to test data are also exhibited in Figure 11, and the statistical results of the ratios of tested to computed values are presented in Table 12. These represent that the rational predictions for tensile strength development of the RAC are given out with these equations. 

## 5. Assessment by Commonly Used Models

For engineering applications, the test results of this study were assessed by commonly used time-dependent models. For the convenience of description and comparison, the symbols with the same meaning are unified and the parameter values are taken as those corresponding to the condition of this study.

The equations proposed by the ACI Committee 209 [55] are,

(10) $$f_{\mathrm{cu}.t}=\frac{t}{4+0.85t}f_{\mathrm{cu},28}$$

(11) $$f_{\mathrm{st}.t}=0.0069{\left[wf_{\mathrm{cu},t}\right]}^{0.5}$$

In Equation (11), *w* is the unit weight of the concrete. In this paper, it takes 2500 kg/m^3^.

The equations proposed by Eurocode 2 and CEB-FIP MC 2010 [56,57] are,

(12) $$f_{\mathrm{cu}.t}=\;\beta_{\mathrm{cc}}(t)f_{\mathrm{cu},28}$$

(13) $$f_{\mathrm{st}.t}=\;f_{\mathrm{st}.28}{\left[\beta_{\mathrm{cc}}(t)\right]}^{2/3}$$

(14) $$\beta_{\mathrm{cc}}(t)=\exp\left\{s\left[1-{\left(\frac{28}{t}\right)}^{0.5}\right]\right\}$$

In Equation (14), *s* = 0.25 for 42.5 strength grade of cement and 0.20 for 52.5 strength grade of cement.

**Figure 12:**
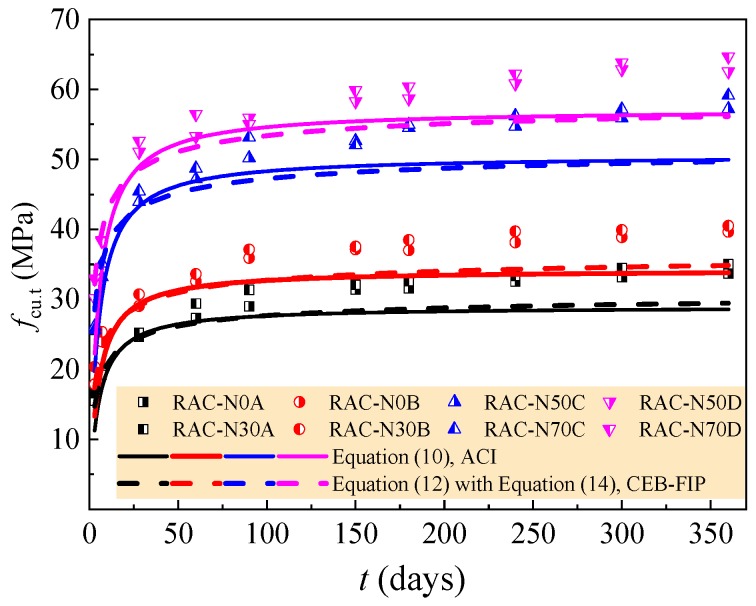
Assessment of compressive strength by time-dependent models of ACI and CEB-FIP.

**Figure 13:**
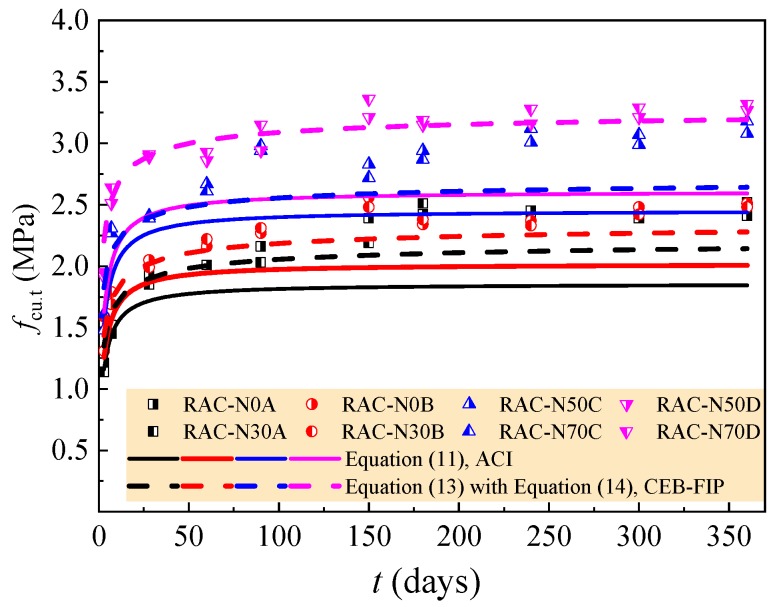
Assessment of the tensile strength by time-dependent models of ACI and CEB-FIP.

The experimental data of the RAC with a rational amount of large particle natural aggregate are assessed by the above equations; the comparison is exhibited in Figure 12 and Figure 13. For the compressive strength of the RAC, almost the same predictions are provided by Equations (10) and (12), and the predictions are conservative, especially after the curing age of 90 days. Relatively, Equation (10) is too conservative with the predicted strength lower than the tested of 24.0–30.3% at 3 days. With the increase of curing age from 28 days to 360 days, the conservative predicted deviations of Equation (10) increase to the maximum of 11.2–16.8%. When the curing age is less than 180 days, Equation (12) provides a good prediction with the conservative predicted deviations within 10%. The predicted deviation increases with the curing age and reaches the maximum of 11.7–14.6% at the curing age of 360 days.

For the tensile strength of the RAC, Equation (11) gives relatively good predictions at the curing age within 28 days. Due to the accumulation of conservative deviation of compressive strength predicted by Equation (10), Equation (11) provides quite a conservative predicted tensile strength of the RAC after the curing age of 90 days. The maximum conservative predicted deviations are 19.6–25.1% at the curing age of 360 days. Comparatively, Equation (13) gives a better prediction than Equation (11) with the maximum predicted deviation of 15.6% at the curing age of 360 days for the RAC with *w*/*c* = 0.60, 0.49 and 0.41, and the best prediction with the maximum predicted deviation within ± 5% for the RAC with *w*/*c* = 0.36. However, it should be noted that the predicted value is 14.1–18.2% higher than the tested one at the curing age of 3 days. This is unsafe when the early strength was used to determine the construction process of the RAC structures.

## 6. Conclusions

The main conclusions of this study are as follows:

(1) The compressive and tensile strength of the new RAC with large particle natural aggregate grew in trends as rapid, fast to steady with an increase of curing age. This was affected by the water/cement ratio and strength grade of the cement. When *w/c* = 0.6 and 0.49, the compressive strength was higher for the RAC with 30% natural aggregate, while the tensile strength was not obviously influenced by the amount of natural aggregate. When *w/c* = 0.41 and 0.36, the compressive and splitting tensile strengths were higher for the RAC with 50% and 70% natural aggregate, while the strengths benefited from 30% natural aggregate. This indicates that the rational amount of large particle natural aggregate existed in the RAC with a different water/cement ratio.
(2) Based on the modification to a predictive model of compressive strength of conventional concrete, the predictive equation of compressive strength of the RAC at any curing age is proposed, in which the main influence factors are the strength and density of cement, the water/cement ratio and the curing age. By introducing the relationship between splitting tensile strength and compressive strength of the RAC at any curing age, the splitting tensile strength can be predicted from the compressive strength at the same curing age. After the fitness analyses, the large prediction can be controlled within 10% over test results. This provides a convenient method for engineering applications.
(3) A rational amount of large particle natural aggregate can be determined to get good strength development. It should be no more than 30% for the RAC in a strength grade no larger than C30 with *w/c* = 0.60 and 0.49, and no less than 50% for the RAC in a strength grade no less than C40 with *w/c* = 0.41 and 0.36. Along with the general prediction of the strength development of all tested RAC, the optimal predictive formulas are proposed for the strength development of the RAC with a rational amount of natural aggregate.
(4) The test results of the RAC with a rational amount of natural aggregate are assessed by the commonly used time-dependent models proposed by ACI and CEB-FIP. These models are conservative to predict the strength development of the RAC, except that the CEB-FIP model provides a higher tensile strength at 3 days.
(5) Due to the lack of studies on the safe prediction of the strength development of RAC with different amounts of large particle natural aggregate, the modern design experimental method needs to be used to estimate numerical mechanical characteristics without the need for real tests.

## Figures and Tables

**Table 1:** Physical and mechanical properties of coarse aggregates.

Coarse Aggregate	RA-N0	RA-N30	RA-N50	RA-N70
Natural aggregate (%)	0	30	50	70
Apparent density (kg/m^3^)	2634.1	2691.5	2732.8	2735.1
Bulk density (kg/m^3^)	1345.8	1410.1	1463.2	1469.7
Close-compacted density (kg/m^3^)	1452.3	1512.4	1600.2	1612.9
Moisture content (%)	3.2	2.1	1.7	0.9
Water absorption of 24 h (%)	5.1	4.7	3.2	1.9
Crushed index (%)	14.7	14.1	13.5	13.2
Silt content (%)	0.42	0.29	0.21	0.19

**Table 2:** Physical and mechanical properties of fine recycled aggregate.

Properties	Values
Fineness modulus	3.5
Apparent density (kg/m^3^)	2395.7
Bulk density (kg/m^3^)	1330
Close-compacted density (kg/m^3^)	1470
Moisture content (%)	5.70
Water absorption of 24 h (%)	9.45
Crush index (%)	9.35

**Table 3:** Physical and mechanical properties of cement.

Grade	Density (kg/m^3^)	Water Requirement of Standard Consistency (%)	Setting Time (min)		Compressive Strength (MPa)		Flexural Strength (MPa)	
			Initial	Final	3 days	28 days	3 days	28 days
42.5	3071	26.9	168	269	28.9	45.2	4.00	5.30
52.5	3132	29.2	142	238	37.1	57.9	6.45	8.64

**Table 4:** Mix proportion of recycled aggregate concrete (RAC).

Mix	*w*/*c*	Natural Aggregate (%)	Cement (kg/m^3^)	Water (kg/m^3^)	Fine RA (kg/m^3^)	Coarse Aggregate (kg/m^3^)		Additional Water (kg/m^3^)
						Natural	Recycled	
RAC-N0A	0.6	0	332	200	651	0	1157	54.9
RAC-N30A	0.6	30	332	200	734	304	710	55.6
RAC-N50A	0.6	50	332	200	736	508	508	50.4
RAC-N70A	0.6	70	332	200	737	712	305	45.3
RAC-N0B	0.49	0	409	200	626	0	1114	52.8
RAC-N30B	0.49	30	409	200	708	293	684	53.6
RAC-N50B	0.49	50	409	200	709	490	490	51.1
RAC-N70B	0.49	70	409	200	711	687	294	43.6
RAC-N0C	0.41	0	435	180	608	0	1119	53.1
RAC-N30C	0.41	30	435	180	688	310	722	53.6
RAC-N50C	0.41	50	435	180	689	517	517	48.3
RAC-N70C	0.41	70	435	180	691	691	311	43.6
RAC-N0D	0.36	0	503	180	612	0	1089	51.2
RAC-N30D	0.36	30	503	180	697	288	674	52.8
RAC-N50D	0.36	50	503	180	698	482	482	47.9
RAC-N70D	0.36	70	503	180	700	676	290	42.9

**Table 5:** Details of test and curing age.

Trial No.	Groups	Designed Curing Age *t* (days)
A, B, C, D	4 × (4 × 10) for compressive strength 4 × (4 × 10) for splitting tensile strength	3, 7, 28, 60, 90, 150, 180, 240, 300, 360

**Table 6:** Compressive strength ratio of the RAC with/without natural aggregate.

RAC	30A/0A	50A/0A	70A/0A	30B/0B	50B/0B	70B/0B	30C/0C	50C/0C	70C/0C	30D/0D	50D/0D	70D/0D
Number	8	8	8	8	8	8	8	8	8	8	8	8
Mean ratio	1.063	0.949	0.954	1.036	0.945	0.944	1.044	1.129	1.158	1.014	1.119	1.146
Variation coefficient	0.044	0.035	0.037	0.042	0.030	0.055	0.036	0.031	0.044	0.025	0.034	0.031

**Table 7:** Splitting tensile strength ratio of RAC with/without natural aggregate.

RAC	30A/0A	50A/0A	70A/0A	30B/0B	50B/0B	70B/0B	30C/0C	50C/0C	70C/0C	30D/0D	50D/0D	70D/0D
Number	8	8	8	8	8	8	8	8	8	8	8	8
Mean ratio	0.998	0.979	1.010	0.993	1.008	1.017	1.007	1.111	1.112	1.029	1.094	1.125
Variation coefficient	0.029	0.046	0.031	0.017	0.031	0.031	0.016	0.029	0.031	0.018	0.034	0.034

**Table 8:** Statistical results of tested data.

RAC trials	A	B	C	D
*k*	0.068	0.071	0.074	0.076
Correlation coefficient	0.954	0.952	0.838	0.830
Standard error	0.005	0.006	0.002	0.005

**Table 9:** General statistical result of the ratios of tested to calculated values.

**RAC-N**	**0A**	**30A**	**50A**	**70B**	**0B**	**30B**	**50B**	**70B**
Number	10	10	10	10	10	10	10	10
Mean ratio	1.02	1.07	0.96	0.97	1.01	1.04	0.94	0.95
Variation coefficient	2.01%	3.32%	4.52%	3.91%	5.24%	2.05%	4.21%	1.89%
Correlation coefficient	0.991	0.987	0.972	0.981	0.982	0.991	0.985	0.993
**RAC-N**	**0C**	**30C**	**50C**	**70C**	**0D**	**30D**	**50D**	**70D**
Number	10	10	10	10	10	10	10	10
Mean ratio	0.88	0.90	1.02	1.06	0.90	0.91	1.02	1.08
Variation coefficient	5.02%	7.24%	4.60%	5.83%	5.69%	6.14%	5.21%	4.47%
Correlation coefficient	0.985	0.971	0.992	0.981	0.961	0.963	0.981	0.985

**Table 10:** Separate statistical result of the ratios of tested to calculated values.

RAC-N	0A	30A	0B	30B	50C	70C	50D	70D
Number	10	10	10	10	10	10	10	10
Mean ratio	0.98	1.02	0.97	1.01	0.97	1.01	0.98	1.02
Variation coefficient	4.21%	3.72%	4.94%	3.11%	3.51%	6.18%	4.66%	5.62%
Correlation coefficient	0.941	0.961	0.972	0.985	0.992	0.972	0.979	0.982

**Table 11:** Statistical results of the general prediction of the splitting tensile strength of RAC.

**RAC-N**	**0** **A**	**30** **A**	**50** **A**	**70** **B**	**0** **B**	**30** **B**	**5** **0** **B**	**7** **0** **B**
Number	10	10	10	10	10	10	10	10
Mean ratio	1.08	1.06	1.05	1.06	1.0	1.02	1.01	0.99
Variation coefficient	9.17%	10.38%	6.79%	9.72%	7.87%	3.16%	8.43%	4.89%
Correlation coefficient	0.989	0.978	0.975	0.978	0.975	0.978	0.924	0.966
**RAC-N**	**0C**	**30C**	**50C**	**70C**	**0D**	**30D**	**5** **0** **D**	**7** **0** **D**
Number	10	10	10	10	10	10	10	10
Mean ratio	0.89	0.92	1.02	1.04	0.96	0.95	1.05	1.06
Variation coefficient	3.81%	4.87%	9.31%	8.34%	5.06%	5.55%	5.71%	6.21%
Correlation coefficient	0.942	0.944	0.959	0.951	0.893	0.933	0.954	0.933

**Table 12:** Statistical results of the accurate prediction of the splitting tensile strength of RAC.

RAC-N	0A	30A	0B	30B	50C	70C	50D	70D
Number	10	10	10	10	10	10	10	10
Mean ratio	1.04	1.02	0.96	0.98	0.96	1.03	0.97	1.02
Variation coefficient	8.35%	9.41%	6.89%	3.69%	8.79%	9.19%	9.33%	7.21%
Correlation coefficient	0.921	0.935	0.951	0.974	0.961	0.955	0.947	0.940

## References

[1] Hendriks C.F., Pietersen H.S. (2000). Sustainable raw materials: Construction and demolition waste–state-of-the-art report of RILEM technical committee 165-SRM.

[2] Li X.P. (2008). Recycling and reuse of waste concrete in China Part I. Material behavior of recycled aggregate concrete. Resour. Conserv. Recycl..

[3] Xiao J.Z., Li W.G., Fan Y.H. (2012). An overview of study on recycled aggregate concrete in China. Constr. Build. Mater..

[4] Lotfi S., Eggimann M., Wagner E. (2015). Performance of recycled aggregate concrete based on a new concrete recycling technology. Constr. Build. Mater..

[5] Kim H., Goulias D.G. (2015). Shrinkage behavior of sustainable concrete with crushed returned concrete aggregate. J. Mater. Civ. Eng..

[6] Otsuki N., Miyazato S., Yodsudjai W. (2003). Influence of recycled aggregate on interfacial transition zone strength, chloride penetration and carbonation of concrete. J. Mater. Civ. Eng..

[7] Li J.H., Xiao H.N., Zhou Y. (2009). Influence of coating recycled aggregate surface with pozzolanic powder on properties of recycled aggregate concrete. Constr. Build. Mater..

[8] Ho N.Y., Lee Y.P.K., Lim W.F. (2013). Efficient utilization of recycled concrete aggregate in structural concrete. J. Mater. Civ. Eng..

[9] Wai H.K., Mahyuddion R., Kenn J.K. (2012). Influence of the amount of recycled coarse aggregate in concrete design and durability properties. Constr. Build. Mater..

[10] Yao Y.F., Jin B.H., Zhang H.G., Lan H.W. (2016). Influence of replacement rate of recycled coarse aggregate on mechanical properties of recycled concrete. J. Guangxi Univ. (Nat. Sci. Ed).

[11] Liu S.X., Wei X.G., Wei W. (2014). Influence of recycled coarse aggregate on recycled concrete performance. Build. Struct..

[12] Seo D.S., Choi H.B. (2014). Effects of the old cement mortar attached to the recycled aggregate surface on the bond characteristics between aggregate and cement mortar. Constr. Build. Mater..

[13] Zhao S.B., Guo Q., Li G.X., Su Y.F., Shao W.J. (2013). Basic mechanical properties of concrete with machine-made sand and recycled coarse aggregate. Appl. Mech. Mater..

[14] Li C.Y., Zhao M.L., Ren F.C., Liang N., Li J., Zhao M.S. (2017). Bond Properties between full-recycled-aggregate concrete and deformed steel bar. Open Civ. Eng. J..

[15] Sidorova A., Vazquez-Ramonich E., Barra-Bizinotto M. (2014). Study of the recycled aggregates natures influence on the aggregate cement paste interface and ITZ. Constr. Build. Mater..

[16] Qiao J.S., Zang P., Huo W.J. (2016). Influence of maximum particle size of coarse aggregate on strength of recycled concrete. J. Hebei United Univ..

[17] Li S.S., Gao D.Y. (2013). Experimental research on the influence of coarse aggregate size on boulder concrete compressive strength. Concrete.

[18] Zhao S.B., Liang N., Liu L.X., Sun L., Yang S. (2011). Experimental study on mechanical properties of fine aggregate concrete made after wet-sieving coarse aggregate. Adv. Mater. Res..

[19] Li X.K., Guo Q., Zhao S.B., Li G.X., Su Y.F. (2013). Mix design and experimental study of fully recycled aggregate concrete. J. North Chin. Univ. Water Resour. Electric Power.

[20] Ministry of Housing and Urban-Rural Construction of the People’s Republic of China (2010). Code for Design of Concrete Structures.

[21] Ministry of Housing and Urban-Rural Construction of the People’s Republic of China (2008). Code for Durability Design of Concrete Structures.

[22] Butler L., West J.S., Tighe S.L. (2013). Effect of recycled concrete coarse aggregate from multiple sources on the hardened properties of concrete with equivalent compressive strength. Constr. Build. Mater..

[23] Fathifazl G., Razaqpur A.G., Isgor O.B., Abbas A., Fournier B., Foo S. (2011). Creep and drying shrinkage characteristics of concrete produced with coarse recycled concrete aggregate. Cem. Concr. Compos..

[24] Corinaldesi V., Moriconi G. (2009). Influence of mineral additions on the performance of 100% recycled aggregate concrete. Constr. Build. Mater..

[25] Elhakam A.A., Mohamed A.E., Awad E. (2012). Influence of self-healing, mixing method and adding silica fume on mechanical properties of recycled aggregates concrete. Constr. Build. Mater..

[26] Kou S., Poon C., Agrela F. (2011). Comparisons of natural and recycled aggregate concretes prepared with the addition of different mineral admixtures. Cem. Concr. Compos..

[27] Brand A.S., Roesler J.R., Salas A. (2012). Initial moisture and mixing effects on higher quality recycled coarse aggregate concrete. Constr. Build. Mater..

[28] Liang Y.C., Ye Z.M., Vernerey F., Xi Y.P. (2015). Development of processing methods to improve strength of concrete with 100% recycled coarse aggregate. J. Mater. Civ. Eng..

[29] Yildirim S.T., Meyer C., Herfellner S. (2015). Effects of internal curing on the strength, drying shrinkage and freeze-thaw resistance of concrete containing recycled concrete aggregates. Constr. Build. Mater..

[30] Cui H.Z., Shi X., Memon S.A., Xing F., Tang W. (2015). Experimental study on the influence of water absorption of recycled coarse aggregates on properties of the resulting concretes. J. Mater. Civ. Eng..

[31] Ismail S., Ramli M. (2014). Mechanical strength and drying shrinkage properties of concrete containing treated coarse recycled concrete aggregates. Constr. Build. Mater..

[32] Guedes M., Evangelista L., de Brito J., Ferro A.C. (2013). Microstructural characterization of concrete prepared with recycled aggregates. Microsc. Microanal..

[33] Evangelista L., Guedes M., de Brito J., Ferro A.C., Pereira M.F. (2015). Physical, chemical and mineralogical properties of fine recycled aggregates made from concrete waste. Constr. Build. Mater..

[34] Liang N., Zhao S.B., Zhu W.W., Yao K.Q., Jin L.H., Qian X.J. (2017). Experimental study on shrinkage behavior of full-recycled aggregate concrete. J. North Chin. Univ. Water Resour. Electric Power.

[35] Evangelista L., de Brito J. (2007). Mechanical behaviour of concrete made with fine recycled concrete aggregates. Cem. Concr. Compos..

[36] Evangelista L., de Brito J. (2010). Durability performance of concrete made with fine recycled concrete aggregates. Cem. Concr. Compos..

[37] Pereira P., Evangelista L., de Brito J. (2012). The effect of superplasticisers on the workability and compressive strength of concrete made with fine recycled concrete aggregates. Constr. Build. Mater..

[38] Pan L.Y., Liang N., Hu F.J., Yao K.Q., Cheng D.D., Pei S.W. (2017). Experimental study and evaluation of mechanical properties of full-recycled coarse aggregate concrete. J. North Chin. Univ. Water Resour. Electric Power.

[39] Yao K.Q., Liang N., Wu H.H., Pei S.W. (2017). Experimental study on strength development of full-recycled-aggregate concrete. Hans J. Civ. Eng..

[40] Li F.L., Li J., Chen S., Zhao W.J. (2011). Experiment of basic mechanical properties of concrete mixed with composite aggregate. Adv. Mater. Res..

[41] Santos S.A., Da Silva P.R., De Brito J. (2017). Mechanical performance evaluation of self-compacting concrete with fine and coarse recycled aggregates from the precast industry. Material.

[42] Li C.Y., Geng H.B., Deng C.H., Li B.C., Zhao S.B. (2019). Experimental investigation on columns of steel fiber reinforced concrete with recycled aggregates under large eccentric compression load. Material.

[43] Cheng D.D., Geng H.B., Li Q., Li B.C., Ma K. (2018). Study on large eccentric compression resistance of steel fiber recycled concrete columns. Hans J. Civ. Eng..

[44] Ding X.X., Li C.Y., Xu Y.Y., Li F.L., Zhao S.B. (2016). Experimental study on long-term compressive strength development of concrete with manufactured sand. Constr. Build. Mater..

[45] Zhao S.B., Ding X.X., Zhao M.S., Li C.Y., Pei S.W. (2017). Experimental study on tensile strength development of concrete with manufactured sand. Constr. Build. Mater..

[46] Ministry of Housing and Urban-Rural Construction of the People’s Republic of China (2011). Recycled Coarse Aggregate for Concrete.

[47] Ministry of Housing and Urban-Rural Construction of the People’s Republic of China (2006). Standard for Technical Requirements and Test Method of Sand and Crushed Stone (or Gravel) for Ordinary Concrete.

[48] Ministry of Housing and Urban-Rural Construction of the People’s Republic of China (2011). Recycled Fine Aggregate for Concrete.

[49] Ministry of Housing and Urban-Rural Construction of the People’s Republic of China (2011). Specification for Mix Proportion Design of Ordinary Concrete.

[50] Ministry of Housing and Urban-Rural Construction of the People’s Republic of China (2002). Standard for Test Method of Mechanical Properties on Ordinary Concrete.

[51] Ministry of Housing and Urban-Rural Construction of the People’s Republic of China (2002). Standard for Test Method of Performance on Ordinary Fresh Concrete.

[52] Larrard F.D. (1999). Concrete Mixture Proportioning: A Scientific Approach.

[53] Li X.K., Ma X.L., Zhang X.Y. (2012). Experimental study on mix proportion of fair-faced concrete for urban bridge. Adv. Mater. Res..

[54] Jin L.H., Ma X.L., Li X.K. (2013). Experimental study on basic properties of fair-faced concrete for urban bridge. Appl. Mechan. Mater..

[55] ACI committee 209 (1992). Prediction of Creep, Shrinkage, and Temperature Effects in Concrete Structures.

[56] PN-EN 1992-1-1, Eurocode 2 (2008). Design of Concrete Structures: Part 1-1: General Rules and Rules for Buildings.

[57] Comite Euro-International Du Beton (2011). CEB-FIP Model Code 2010.

